# Pedicle morphometry in patients with adolescent idiopathic scoliosis

**DOI:** 10.4103/0019-5413.62084

**Published:** 2010

**Authors:** Bidre Upendra, Devkant Meena, Pankaj Kandwal, Abrar Ahmed, Buddhadev Chowdhury, Arvind Jayaswal

**Affiliations:** Department of Orthopaedics, All India Institute of Medical Sciences, Ansari Nagar, New Delhi, India

**Keywords:** Adolescent idiopathic scoliosis, chord length, pedicle morphometry, transverse pedicle angle, transverse pedicle width

## Abstract

**Background:**

The key to the safe and effective use of thoracic pedicle screws in the deformed spine is to thoroughly understand pedicle anatomy. There are a few studies related to pedicle anatomy in the Indian population and no pedicle morphometric studies in scoliosis patients. The present study aims to highlight the differential features of pedicle morphometry, including pedicle width, transverse pedicle angle and the depth to anterior cortex on the concave and convex side, in a group of Indian patients with adolescent idiopathic scoliosis and compare this to that of a western population.

**Materials and Methods:**

This is a prospective study of 24 patients with adolescent idiopathic scoliosis. The average age is 14.6 years (12.3-18.3 years) of which 14 were females and 10 were males. All the patients underwent CT scan using Siemens 4^th^ generation scanner. The scans were analyzed by measuring the transverse pedicle width, transverse pedicle angle and the chord length; all the measurements being made both on the convex as well as the concave pedicle. Statistical analysis was performed with unpaired ‘t’ test.

**Results:**

A total of 1295 measurements were performed from 24 patients and an average of 215 pedicles were assessed for each set of the measurements made. The transverse pedicle width was consistently found to be smaller on concave side in comparison with the convex side at all levels except at T1. The transverse pedicle angle was greater on the concave side at all levels as compared to the convex side, though there was wide individual variation. The depth to anterior cortex was lesser on convex side in comparison to the concave side except at T1.

**Conclusions:**

The concave pedicle is much thinner and directed more medially than the convex side, especially at the apical region of the scoliotic curve. The pedicle anatomy in scoliosis patients shows very high individual variations and a careful study of pre-operative CT scans is essential for planning proper pedicle screw placement. Slightly longer screws can be accommodated on the concave side as compared to the convex side, though the difference in the chord length is not statistically significant at most levels.

## INTRODUCTION

Scoliosis is now widely recognized as a three dimensional deformity of the spine with lateral deviation in the coronal plane, lordosis in the sagittal plane and rotation in the axial plane, particularly pronounced in the apical vertebrae. Surgical management of Adolescent Idiopathic Scoliosis (AIS) has evolved from the era of Harrington instrumentation to the use of segmental thoracic pedicle screws, which has now become the most popular method to get good three dimensional correction of the deformity. The key to the safe and effective use of segmental thoracic pedicle screws in the deformed spine is to thoroughly understand pedicle anatomy. Studies have been done on pedicle morphometry in non-deformed thoracic and lumbar spine in order to find a consistent pattern that can be applied while performing pedicle screw placement during surgery.^1^–^13^ Studies have shown that the transverse pedicle width is the least diameter of the pedicle at any given level and is the limiting factor in using the largest diameter screw. Further, interracial pedicle anatomy has been shown to be highly variable by many studies.^2^,^4^–^6^,^11^–^13^ There are anatomical changes, like deviation of the body towards the concave side, shorter and thinner pedicles on the concave side and longer and thicker pedicles on the convex sides in the vertebral body and pedicles of the scoliotic vertebrae, most pronounced in the apical region. There are a few studies on pedicle anatomy in the Indian population^3^,^6^,^7^ and none in the Indian scoliosis patients. The present study aims to highlight the differential features of pedicle morphometry including the pedicle width, transverse pedicle angle and the depth to anterior cortex on the concave and convex side in a group of Indian patients with AIS and to compare this to that of a western population as reported in the literature.

## MATERIALS AND METHODS

**Figure 1:**
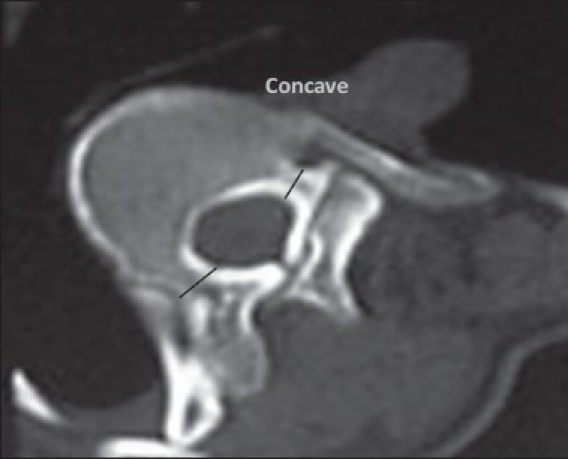
Axial CT image depicting transverse pedicle width

**Figure 2:**
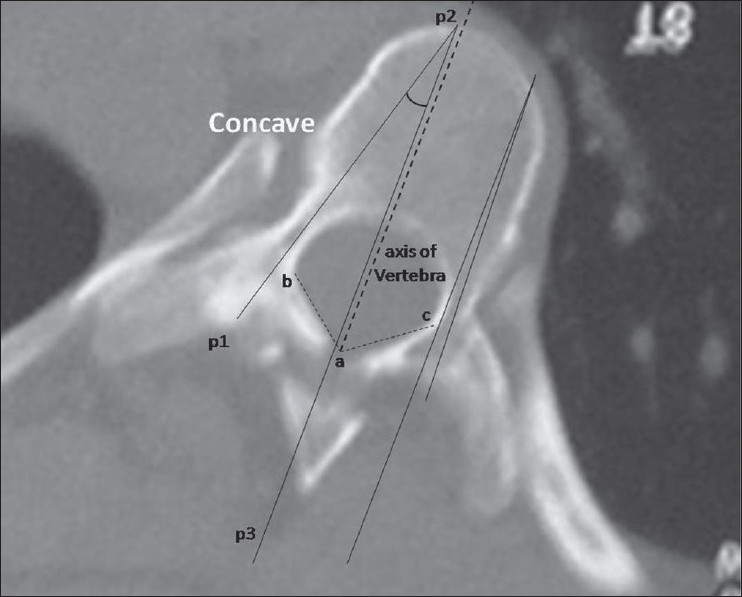
Axial CT image depicting transverse pedicle angle-Concave and convex sides with vertebral axis. It is the angle between the pedicle axis and a line parallel to the vertebral axis in the transverse plane. The vertebral axis is the line bisecting the angle between the lines formed by joining the junction of both laminae posteriorly (a) and one point each on the medial cortical wall where the lamina meets the pedicle (b and c). The transverse pedicle angle is formed by the angle between the pedicle axis p1-p2 and a line parallel to the vertebral axis p2-p3 on the concave side

**Figure 3:**
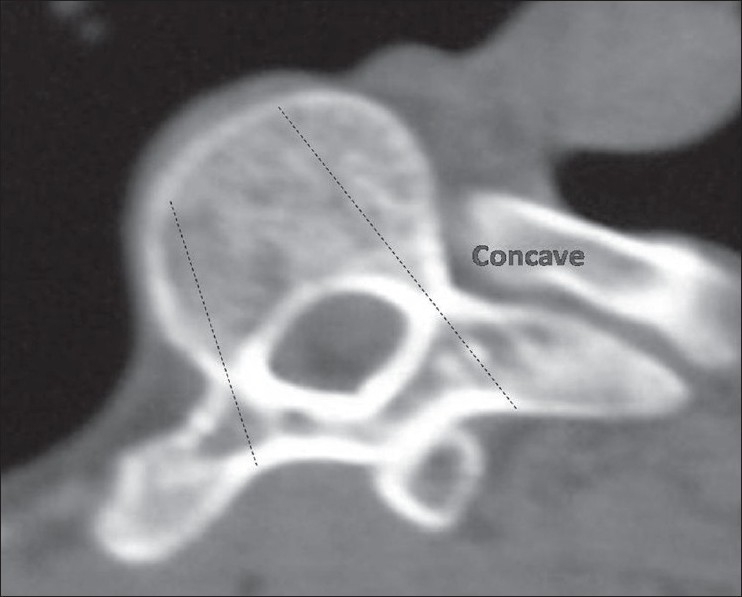
Axial CT image depicting depath to anterior cortex/chord length-Concave and convex sides

A prospective cohort study was conducted at our institute among the patients attending the scoliosis clinic from April 2003 to June 2007. Twenty four patients having adolescent idiopathic scoliosis, with curves less than 100° were included in the study. The average age was 14.6years (12.3-18.3 years) of which 14 were females and 10 males. All patients had right thoracic/ thoracolumbar curves (12 had King II, 7 had King III and 5 had King IV curves). All the patients underwent CT scan using Siemens 4^th^ generation scanner. A lateral topogram was taken in all the patients and 3 mm axial cuts were taken through the region of the pedicles for morphometric analysis thus minimizing the radiation exposure. Written consent was obtained from all patients included in the study and the study protocol was approved by the institute ethics committee. The scans were analyzed by measuring the transverse pedicle width, transverse pedicle angle and the chord length at both on the convex as well as the concave pedicle. It is important to get the CT scan sections at right angles to the corresponding pedicles in order to be able to make the measurements accurately. The apical vertebra is always oriented in the horizontal plane and is easier to get sections at right angles to it, however the ends of the curve have the most tilted vertebrae and it is tedious to get scan sections at their right angles and this tilt increases with more severe curves. For this purpose, very severe curves with more than 100° cobbs were not included in the study. The study specifically included the three most important anatomical parameters of the pedicle useful during the free-hand insertion of thoracic pedicle screws in scoliotic spine.These are:

1. Transverse pedicle width-This provides the surgeon the diameter of the largest pedicle screw that can be safely used at a given level. It is defined as the narrowest width of the pedicle in the transverse plane. For ease and reproducibility, outer cortex to outer cortex distance was measured on the CT scans. Radiographic measurements were converted to actual dimensions by using the scale provided with each CT scan [Figure 1].
2. Transverse pedicle angle-This gives the surgeon the amount of transverse angulation required while making the pilot hole for pedicle screw insertion. It is defined as the angle between the pedicle axis and a line parallel to the vertebral axis in the transverse plane [Figure 2]. The vertebral axis is the line bisecting the angle between the lines formed by joining the junction of both laminae posteriorly(a) and one point each on the medial cortical wall where the lamina meets the pedicle (b and c). The transverse pedicle angle is formed by the angle between the pedicle axis p1-p2 and a line parallel to the vertebral axis p2-p3 on the concave side [Figure 2].
3. Depth to anterior cortex/Chord length: This gives the appropriate length of the screw that can be used at any given level. It is defined as the distance along the transverse pedicle axis from the most posterior point on the transverse process to the anterior cortex of the vertebra [Figure 3].

All the parameters on the concave side were compared with the corresponding value on the convex side. Statistical analysis was performed with unpaired ‘t’ test. Difference was regarded as significant when a value was below *P*<0.05. The angle measurements were made directly from the bone window images using computer software for angle measurements. The distances were measured from digital caliper (Mitutoyo Company, Japan) on the CT films with accuracy of 0.01 mm.

## RESULTS

A total of 1295 measurements were made from 24 patients; an average of 216 pedicles were assessed for each set of the measurements made. There were 213 and 223 measurements made for pedicle width on the convex side and the concave sides respectively; similarly for transverse pedicle angle, 217 and 216 measurements were made on the convex side and the concave sides respectively, and for chord length, 213 measurements were made on both sides. All the measurements were not possible in a given section of CT scan and hence there is difference in the number of measurements made for each set of measurements. The average Cobbs angle was 76° (58°-98°). All patients had a major right thoracic/ thoracolumbar curve (12 had King II,7 had King III and 5 had King IV curves).

### Transverse pedicle width

The transverse width was consistently found to be smaller at all levels on the concave pedicle as compared to the corresponding convex pedicle, except at T1 [Table 1]. However, the differences between concave and convex sides were not statistically significant at most of the levels (*P*>0.05) except at T3,T6,T7 and L5 (*P*<0.05).The least average value on the concave side was at the T3 Pedicle (4.1 mm) and on the convex side at T4 pedicle (4.92 mm). The maximum transverse diameter was found at L5 on both the side. It is also evident from the table that as one moved down the spine, the pedicle width decreases from T1 to T3-T4 and subsequently increases with each level reaching the maximum at L5, both on concave and convex sides [Table 1].

**Table 1:** Transverse pedicle width (a) Convex pedicle (b) Concave pedicle

a) Convex pedicle					(b) Concave pedicle				
	
Level	No. of pedicles	Mean width (mm)	Range (mm)	S.D	Level	No. of pedicles	Mean width (mm)	Range (mm)	S.D
T1	12	5.1	3.8-7.1	1.76	T1	12	5.2	3.6-6.8	1.01
T2	10	5.35	4.1-7.2	1.25	T2	11	5.2	3.7-7.2	1.36
T3	13	5.12	3.6-6.8	1.11	T3	13	4.1	3.4-5.1	0.31
T4	14	4.92	3.0-5.8	0.86	T4	15	4.42	3.6-5.0	0.62
T5	9	5.87	5.1-7.2	0.61	T5	9	5.2	4.1-6.2	0.41
T6	11	6.6	5.4-8.1	1.14	T6	13	4.95	4.0-6.1	0.56
T7	9	6.60	5.1-9.2	1.32	T7	11	5.50	4.6-6.3	0.21
T8	14	5.35	4.5-6.3	0.68	T8	14	5.33	4.2-6.4	0.46
T9	12	5.08	4.3-6.2	0.89	T9	12	4.96	3.8-6.1	0.65
T10	15	5.36	4.2-7.7	1.12	T10	15	5.21	4.1-7.1	1.01
T11	16	6.27	4.8-8.2	1.36	T11	16	5.60	3.6-8.1	1.65
T12	19	6.8	5.0-9.7	1.33	T12	22	5.22	4.1-7.2	0.89
L1	18	6.48	4.6-10.1	1.78	L1	19	6.04	4.0-9.2	1.63
L2	13	5.8	4.5-9.0	1.14	L2	13	6.16	4.6-9.1	1.06
L3	11	7.77	6.1-10.3	1.37	L3	11	7.46	5.1-10.3	1.83
L4	9	8.9	7.2-12.1	1.22	L4	9	8.16	6.1-10.4	1.71
L5	8	12.45	11.4-14.8	0.96	L5	8	9.85	8.1-12.0	1.44

### Transverse pedicle angle

The transverse pedicle angle was higher on the concave side at all levels compared to the corresponding convex pedicles. However, there was wide individual variation (see range and S.D. in Table 2) and the differences between the convex and the concave sides were not statistically significant at most levels (*P*>0.05) except T6, T7 and L5. Further, as one moved down the spine there was no consistent pattern observed as found in the non-deformed spine^1^, where the transverse pedicle angle is known to gradually decrease from T1 to T12 and then again increases from L1 to L5. In contrast the maximum transverse angle was noted at the T7 concave pedicle, which was most often the apical region of the major thoracic curve.

**Table 2:** Transverse pedicle angle; (a) Convex pedicle (b) Concave pedicle

(a) Convex pedicle					(b) Concave pedicle				
	
Level	No. of pedicles	Mean angle (Deg.)	Range (Deg.)	S.D	Level	No. of pedicles	Mean angle (Deg.)	Range (Deg.)	S.D
T1	9	20.67	16-28	4.66	T1	9	21.33	15-30	5.63
T2	11	17.67	14-25	4.96	T2	13	20.7	16-27	4.36
T3	13	12.67	7-21	6.63	T3	11	16	9-20	4.23
T4	16	14.6	0-20	8.14	T4	15	17.75	5-25	5.32
T5	9	18.25	10-26	5.1	T5	9	19.67	10-29	8.65
T6	13	14.5	4-24	5.96	T6	13	20.75	13-34	9.11
T7	11	17	15-20	2.33	T7	11	26.0	20-31	1.14
T8	14	15	5-26	4.69	T8	17	15.67	3-28	9.68
T9	12	15.33	10-18	2.89	T9	12	19.5	14-22.5	3.34
T10	15	12.4	7-19	3.66	T10	15	12.8	4-24	8.24
T11	16	11	3-19	6.93	T11	16	12.0	4-18	6.52
T12	19	13.56	0-30	9.98	T12	16	14.8	5-24	6.59
L1	19	16.38	0-26	8.74	L1	19	16.6	4-26	6.56
L2	13	16.75	5-35	9.66	L2	13	17.6	4-23	5.18
L3	11	12.8	5-21	5.73	L3	11	16	8-23	6.21
L4	9	17.8	18-24	2.66	L4	9	18.7	12-24	4.43
L5	7	18	15-22	2.12	L5	7	24	19-28	4.98

### Depth to anterior cortex along pedicle axis or chord length

The depth to anterior cortex is smaller in the upper thoracic spine and increases as we move down to the lumbar spine. It was also observed that the chord length on the convex side is lesser in comparison to the corresponding concave side except at T1 [Table 3]. However, the differences were again found to be statistically insignificant (*P*>0.05) at most levels except at T3, T10 and L3 (*P*<0.05).

**Table 3:** Depth to anterior cortex along pedicle axis or chord length (a) Convex pedicle (b) Concave pedicle

(a) Convex pedicle					(b) Concave pedicle				
	
Level	No. of pedicles	Mean depth (mm)	Range (mm)	S.D	Level	No. of pedicles	Mean depth (mm)	Range (mm)	S.D
T1	12	29.75	25.1-32.3	2.31	T1	12	27.67	25.1-32.6	2.89
T2	10	30.5	27.2-32.3	2.38	T2	10	32.83	28.1-37.3	3.55
T3	13	32.8	18.1-41.3	6.59	T3	13	37.33	32.1-40.5	3.89
T4	14	34.83	30.1-37.5	2.11	T4	14	35.75	30.4-40.5	4.12
T5	9	35.33	32.2-38.6	1.96	T5	9	37.0	32.1-43.1	4.22
T6	11	38.5	30.8-43.2	4.66	T6	11	39.1	37.1-43.2	3.11
T7	9	41.5	38.2-43.1	1.95	T7	9	41.8	39.2-43.8	2.12
T8	14	40.2	38.5-41.6	1.86	T8	14	42.1	40.0-44.8	1.68
T9	12	43.5	37.1-53.2	4.96	T9	12	44.2	35.4-46.5	4.97
T10	15	33.35	28.4-41.2	4.22	T10	15	41.5	38.1-46.8	3.12
T11	16	37.5	35.1-42.6	1.69	T11	16	39.5	35.1-46.1	4.2
T12	19	40.27	32.5-47.1	5.86	T12	19	42.2	28.9-50.4	5.66
L1	18	41.46	35.4-52.6	4.39	L1	18	46.8	36.1-55.7	5.76
L2	13	47.37	32.1-54.8	4.96	L2	13	49.86	43.2-54.8	4.11
L3	11	48.56	42.5-52.9	3.64	L3	11	51.2	45.1-55.4	3.21
L4	9	46.2	44.5-47.6	2.14	L4	9	47.65	45.1-50.9	2.11
L5	8	47.0	45.2-50.8	2.08	L5	8	48.2	45.1-50.8	2.89

**Figure 4:**
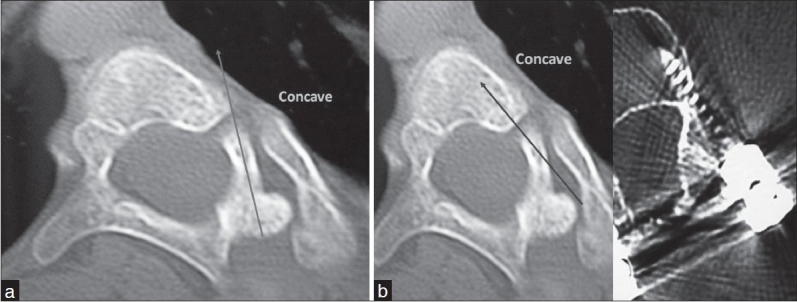
(a) Curved pedicle at the apical vertebra.Trans-pedicular screws perforate the lateral wall of the body. (b) In-out-in technique for placement of screws within the vertebral body; Post-op CT of in-out-in screw

There was no significant difference found between the male and female patients in any of the above parameters. Apart from these measurements we also observed curving of the pedicles especially in the apical region. This was observed in apical vertebra of two patients with Cobb's angle of >80° [Figure 4a and b].

## DISCUSSION

Clinical studies have shown acceptable safety for use of pedicle screws in scoliosis patients.^14^–^17^ The single most important anatomical parameter that limits the use of pedicle screws in the thoracic spine is the transverse pedicle width. In the non-deformed spine the pedicle width is least between T3 to T6 region of the spine.^1^,^5^,^6^,^8^ ^18^ The pedicles in the scoliotic spine have been shown to be thinner on the concave side as compared to the convex side.^14^,^19^,^20^ The present study reinforces these findings and the concave pedicle was found to be significantly thinner than the convex pedicle at T3,T6,T7 and L5 (*P*<0.05). However, the thinnest pedicles were measured at T3-T4 levels and not at the apex of the deformity. In their multiplanar CT study on scoliosis patients, Katsushi *et al*,^20^ also found that the thinnest pedicles are between T3-T5 on the concave side. In contrast, Liljenquest *et al,*^19^ found the thinnest pedicles in the concave apical region of the thoracic curves between the T7 and T10, but their study included vertebrae from T5 to L4 only [Table 4].

**Table 4:** Comparison of pedicle width and transverse pedicle angle in Western, Japanese and Indian patients; (a) Mean pedicle width; (b) Mean transverse pedicle angle

(a) Mean pedicle width (mm)						

	Convex pedicle			Concave pedicle		
		
	Liljenqvist spine 2000 endosteal width	Katsushi Takeshita spine 2009 outer pedicle width	Present study outer pedicle width	Liljenqvist spine 2000 endosteal width	Katsushi Takeshita spine 2009 outer pedicle width	Present study outer pedicle width
T1	-	6.3	5.1	-	6.0	5.2
T2	-	5.5	5.35	-	4.9	5.2
T3	-	4.7	5.12	-	3.3	4.1
T4	-	4.3	4.92	-	2.7	4.42
T5	3.7	4.5	5.87	3.7	3.0	5.2
T6	-	4.4	6.6	-	3.5	4.95
T7	4.1	4.6	6.60	3.1	3.7	5.50
T8	4.2	4.6	5.35	2.5	3.8	5.33
T9	4.2	4.9	5.08	3.3	4.1	4.96
T10	5.0	6.0	5.36	4.2	5.2	5.51
T11	5.7	7.2	6.27	5.4	6.7	5.60
T12	5.9	7.1	6.8	5.2	6.8	5.22
L1	5.1	5.6	6.48	5.1	5.8	6.04
L2	4.8	5.9	5.8	5.3	6.3	6.16
L3	7.0	7.4	7.77	6.7	7.8	7.46
L4	9.4	8.7	8.9	9.5	9.0	8.16
L5	-	10.0	12.45	-	9.1	9.85


**(b) Mean transverse pedicle angle (Degrees)**						

	**Convex pedicle**			**Concave pedicle**		
		
	**Liljenqvist spine 2000**	**Katsushi Takeshita spine 2009**	**Present study**	**Liljenqvist spine 2000**	**Katsushi Takeshita spine 2009**	**Present study**


T1	-	26.9	20.67	-	27.1	21.33
T2	-	17.3	17.67	-	17.4	20.7
T3	-	9.4	12.67	-	13.5	16
T4	-	7.1	14.6	-	8.4	17.75
T5	11.7	5.2	18.25	11.7°	7.1	19.67
T6	-	5.0	14.5	-	4.0	20.75
T7	10	3.3	17	9.8	4.3	26.0
T8	10.5	2.2	15	9.7	4.6	15.67
T9	9.9	4.4	15.33	9.5	4.0	19.5
T10	8.9	3.7	12.4	9.3	5.8	12.0
T11	7	4.9	11	6.4	6.3	12.0
T12	7.8	5.8	13.56	6.6	4.6	14.8
L1	9.2	8.0	16.38	8.9	7.9	16.0
L2	10.9	9.7	16.75	10.6	10.6	17.6
L3	12.3	13.6	12.8	11.3	13.1	16
L4	12.2	14.8	21	11.5	15.1	16.7
L5	-	22.5	18	-	24.5	24

As with our study, both these studies also did not find any significant differences between the male and female patients. In comparison with the studies in the non- deformed spine^1^,^5^,^8^ the scoliotic spine shows a similar pattern of transverse pedicle width decreasing from T1 to T4 and then gradually increasing from T4 downwards. This has also been shown to be true for non-scoliotic Indian patients.^6^

The other issue regarding the pedicle width concerns the absolute values of the pedicle screw diameter that can be safely used. The study of Liljenqvist *et al*,^19^ gives the endosteal diameter of the pedicle isthmus with values as low as 2.5 mm at the concave pedicle of apical vertebra (T8). However, the thickness of the pedicular cortex measures approximately between 1 and 2 mm. Therefore, these values would increase by 1-2 mm for the outer diameter of the pedicle and it has been shown that pedicle screws can be safely inserted up to 80%^21^ to 115%^22^ of the outside pedicle diameter without compromise to the pedicle structure or fixation.

The study by O'Brien *et al*,^14^ shows that the least concave pedicle outside diameter of about 5.7 mm. Therefore a 5.5 mm-6 mm screw can be passed through these pedicles with expansion of pedicle in adolescents.^22^ The Japanese study^20^ on scoliosis patients and the present study show much smaller pedicles in these populations. The outside least diameter was 2.7 mm in the Japanese population and 4.1 mm in the present study. This may indicate that a shorter stature would result in thinner pedicles (thinnest in Japanese, intermediate in Indians and thicker in western population).

Further, if we look at the pedicle width in the non-scoliosis patients, the values in the Asian population^2^,^4^,^5^ including an Indian study,^6^ are about 1-2 mm lesser than the values quoted in studies on western population. Therefore, it may be advisable to use screws with 0.5 to1 mm lesser diameter in Indian patients than the values quoted in western studies.

The second important anatomical parameter to be considered during pedicle screw insertion is the transverse pedicle angle. The present study shows that transverse pedicle angle was higher on the concave side at all levels compared to the corresponding convex pedicles. In contrast, Liljenquest *et al*,^19^ have reported that the convex pedicle angle was consistently more than the concave angulation at all levels [Table 4]. However, they have pointed out that the data on transverse pedicle angulation showed the greatest variations among the values given in their study.

The observations of the present study are consistent with the findings of Katsushi *et al*^20^ [Table 4], who have reported that the concave pedicle angulation in the transverse plane to be more than the convex pedicle angulation. The reason for concave pedicle to be more angulated could be due to the intravertebral deformation that develops with rotation of the scoliotic spine.^23^ This also explains the finding in the present study that the difference between concave and convex pedicle angulation was statistically significant only in the apical region of the curve (T6, T7). However, the transverse pedicle angulations in the present study also showed high individual variation (as was observed by Liljenquest *et al.* 19) and failed to show the pattern followed in the non-scoliotic spine.^1^,^5^ ^6^,^8^ Both Asian^2^,^5^,^6^ and western^1^,^8^ studies, in the non-deformed spine, have shown that the transverse pedicle angle consistently decreases from a maximum at T1 (25-35°) to zero or negative angulation at T12. In contrast the present study shows the maximum transverse pedicle angle was observed in the periapical region with the maximum average transverse angle recorded at the T7 concave pedicle.

O'Brien *et al*,^14^ in their study on AIS patients have pointed out that the periapical vertebrae show “wind swipe deformity” with curving of the pedicles. They have suggested that this does not interfere with the passage of pedicle screws at these levels. In the present study, we found that there was pedicle curving in the apical region of 2 patients with both patients having a Cobb's angle of >80°. In contrast to the views of O'Brien *et al*,^14^ we feel that an attempt to put a trans-pedicular screw with the pedicle curvature would lead to lateral perforation of the body [Figure 4a]. Also, it is advisable to look for such deformed pedicles as the severity of the scoliotic curve increases (Cobbs>80-90°). It is wise to resort to “in-out-in” technique^15^ of screw placement when one recognizes such deformed pedicles on the pre-operative CT scan [Figure 4b]. The ‘in-out-in’ technique^15^ offers 170% more width for screw insertion than the transpedicular insertion and the strength of the screw is about 70-80% of the transpedicular screws, which is better than the laminar hooks.

The third parameter of importance for selecting the appropriate pedicle screw length is the vertebral chord length (depth to anterior cortex along pedicle axis). Our study shows that the concave side can accommodate a slightly longer pedicle screw than the corresponding convex side. However, the increase in the concave chord length in comparison with the convex side was minimal and statistically insignificant at most levels. Katsushi *et al,*^20^ and Liljenquest *et al*,^19^ have also reported on the longer chord length on the concave pedicle of the scoliotic curve as compared to the convex side. The most probable reason for this finding is the intravertebral deformation reported by Tomasz *et al*,^23^. They have shown in their study that the vertebral body drifts towards the concavity in the transverse plane with the rotation of the spine. Consequently, as vertebral body is slightly shifted to the concave side, a longer chord length is observed on the concave side.

The present study is limited as it has included only the important transverse plane parameters required during free-hand insertion of pedicle screws in a scoliotic spine. The sagittal parameters, which have not been included in this study, are of lesser importance in thoracic pedicle screw insertion (sagittal width is always more than the transverse width). No cadaver measurements were made from any bone specimens during this study. However, a good correlation has been shown between CT-based and true morphometric measurements of vertebral and pedicular morphometry in cadavers.^8^,^17^,^21^ Also, Xiong *et al*,^17^ have demonstrated that a tilt of less than 10° does not affect the accuracy of CT-based measurements of the pedicles in the horizontal plane.

This study concludes that while planning pedicle screws in a scoliotic spine the surgeon should be aware of the fact that the concave pedicle is much thinner and directed more medially than the convex side, especially at the apical region of the scoliotic curve. Also, one should be aware of the intravertebral deformity and the curving of pedicles in the apical region (windswipe deformity) as the magnitude of the curve increases. The pedicle anatomy in scoliosis patients shows very high individual variations and a careful study of pre-operative CT scans is essential for planning proper pedicle screw placement. Further, in comparison with the western population, the Indian patients have smaller transverse pedicle widths and higher transverse pedicle angles. With regard to the length of the pedicle screws, slightly longer screws can be accommodated on the concave side as compared to the convex side, though the difference in the chord length is not statistically significant at most levels.
